# Sex- and growth-specific characteristics of small for gestational age infants: a prospective cohort study

**DOI:** 10.1186/s13293-020-00300-z

**Published:** 2020-05-05

**Authors:** Eva R. van der Vlugt, Petra E. Verburg, Shalem Y. Leemaqz, Lesley M. E. McCowan, Lucilla Poston, Louise C. Kenny, Jenny Myers, James J. Walker, Gustaaf A. Dekker, Claire T. Roberts, on behalf of the SCOPE Consortium

**Affiliations:** 1 The Robinson Research Institute and Adelaide Medical School, University of Adelaide, Adelaide, Australia; 2 Adelaide Medical School, University of Adelaide, Adelaide, Australia; 3 The VU University Amsterdam, Amsterdam, the Netherlands; 4 The University of Groningen, Groningen, the Netherlands; 5 The Women and Children’s Division, Lyell McEwin Hospital, University of Adelaide, Adelaide, Australia; 6 The Department of Obstetrics and Gynaecology, University of Auckland, Auckland, New Zealand; 7 The Division of Woman’s Health, Women’s Health Academic Centre, King’s College London and King’s Health Partners, London, UK; 8 Department of Women’s and Children’s Health, Institute of Translational Research, Faculty of Health and Life Sciences, University of Liverpool, Liverpool, UK; 9 Maternal and Fetal Health Research Centre, University of Manchester, Manchester, UK; 10 Section of Obstetrics and Gynaecology, Institute of Biochemical and Clinical Sciences, University of Leeds, Leeds, UK; 11 Flinders Institute of Health and Medical Research, Flinders University, Bedford Park, Australia

**Keywords:** Small for gestational age, Sexual dimorphism, Risk factor, Asymmetric growth, Symmetric growth

## Abstract

**Background:**

Asymmetric fetal growth and male sex are both associated with adverse neonatal outcome. However, less is known about the influence of asymmetric growth and fetal sex within SGA neonates, a group of infants already at increased risk for adverse neonatal outcomes. The aim of the present study was to provide insight into variance in risk factors for SGA in a fetal sex- and growth symmetry-specific way.

**Methods:**

For this prospective, multicenter cohort study, data from the Screening for Pregnancy Endpoints (SCOPE) study were used with 5628 nulliparous participants, of which 633 (11.3%) pregnancies were complicated with SGA and 3376 (60.0%) women had uncomplicated pregnancies. Association between risk factors for SGA, SGA subgroups, and uncomplicated pregnancies were assessed with multivariable analyses.

**Results:**

Prevalence of asymmetric growth varied from 45.8% of SGA infants to 5.5% of infants with a customized birthweight > 90th percentile (*p* < 0.001). Significantly more SGA males had asymmetric growth compared to SGA female infants (51.2% vs 40.4%, *p* = 0.009). Maternal pre-pregnancy diet and BMI < 20 and ≥ 30 were significantly associated with symmetric SGA but not with asymmetric SGA. Asymmetric SGA infants had not only lower customized birthweight percentile (4.4 (SD 2.8) vs 5.0 (SD 3.0), *p* < 0.001), but also lower rates of stillbirth (*p* = 0.041) and less often Apgar scores < 7 (*p* = 0.060).

**Conclusions:**

Among SGA infants, low customized birthweight percentiles and male sex are associated with asymmetric growth. Only symmetric SGA is significantly associated with maternal risk factors in early pregnancy. There is a substantial variance in risk factors and neonatal outcomes for SGA based on growth symmetry, implying a different pathogenesis.

**Trial registration:**

ACTRN12607000551493

## Background

Small for gestational age (SGA) can be defined as neonates with a birthweight below the < 10th percentile customized for maternal factors such as parity, weight, height, and ethnicity [1–3]. SGA is associated with increased rates of stillbirth and neonatal death as well as metabolic disease in later life [1, 4–6]. SGA has many different causes and the aetiology of ‘being SGA’ in this heterogeneous group of infants is not yet understood [7–9]. Current risk prediction for SGA, including maternal risk factors, biomarkers, and ultrasound measurements is insufficient to reliably predict SGA and in clinical practice less than half of SGA infants are usually recognized before birth [7, 9–12].

Fetal growth restriction (FGR) implies the failure of a fetus to achieve its growth potential by showing reduced growth on serial ultrasound evaluation. In *The Lancet’s* Stillbirths Series, Bhutta et al. estimated that improved detection and management of FGR could reduce stillbirth rates by 20% [13]. Early detection of FGR may benefit from closer monitoring and early intervention, although methods of monitoring FGR are improving, current methods are not yet reliable [14]. Although both FGR and SGA are associated with increased rates of stillbirth and adverse perinatal outcome, not all FGR will result in a SGA infant as the birth weight may be restricted but not below the designated customized birthweight percentile [3, 14, 15]. Among growth restricted fetuses and subsequently neonates, a distinction can be made between infants with a birth length or head circumference that is either proportional (symmetric) versus disproportional (asymmetric) to the infant’s weight [16–19]. Previous studies have shown that asymmetric infants are at increased risk for neonatal death, operative interventions and respiratory distress compared to symmetric infants [4, 15, 17–20].

In addition to the type of growth restriction, fetal sex is also known to influence pregnancy and neonatal outcome. While male-bearing pregnancies are at increased risk for early preterm birth, (term) preeclampsia and acute fetal distress, and also have higher rates of caesarean sections, female infants are more likely to be growth restricted but have fewer complications during and after birth [21–24]. In light of these observations, Clifton et al. described differences in growth reduction between male and female fetuses in response to an adverse environment in utero [23]. Whereas female fetuses reduce growth during maternal stress, males continue to grow thereby placing themselves at increased risk for stillbirth and neonatal death [23, 25].

Asymmetric fetal growth and fetal sex are both known to be associated with neonatal outcomes, less is known about the influence of asymmetric growth and fetal sex within SGA neonates, a group of infants already at increased risk for adverse neonatal outcome [1, 4–6]. New insights into these different SGA subgroups could contribute to an improved understanding of its aetiology and inform new methods for more reliable SGA risk prediction. Therefore, the aim of the present study was to provide insight into differences in risk factors for SGA in a fetal sex- and growth symmetry-specific way.

## Methods

### Study protocol

Data from the Screening for Pregnancy Endpoints (SCOPE) study were used. In short, the SCOPE study was a prospective, multicenter cohort study with the main aim to develop screening tests to predict preeclampsia, spontaneous preterm birth and SGA infants. The SCOPE study had recruitment sites in Auckland (New Zealand), Adelaide (Australia), Manchester, Leeds, London (UK), and Cork (Ireland) and recruited participants between 2004 and 2011. Nulliparous women with a singleton pregnancy less than 16 weeks of gestation were eligible for the study. Women with major risk factors for preeclampsia, SGA and spontaneous pre-term birth were excluded from the study (e.g., chronic hypertension requiring antihypertensive drugs, pre-existing diabetes, antiphospholipid syndrome, ≥ 3 abortions or miscarriages, cervical suture, known fetal anomaly). Detailed information about in- and exclusion criteria are described elsewhere [26]. Ethical approval was obtained from the local institutional ethics committees and all participants gave written informed consent.

Participants were interviewed and examined by a research midwife at 15 ± 1 weeks’ gestation. This interview included information about demographics, medical history of both participant and family, as well as information about the current pregnancy including: vaginal bleeding, diet, use of supplements and medication, smoking, alcohol and recreational drug use for both the 3 months before and after becoming pregnant. Weight, height and blood pressure were measured. Maternal socio-economic index (SEI) score was estimated [27]. At the appointments at 15 ± 1 weeks’ and 20 ± 1 weeks’ gestation, participants completed the Edinburgh Postnatal Depression Scale, the Short form State-Trait Anxiety Inventory and Perceived Stress Scale [28–30]. Morphology ultrasonography, including uterine and umbilical Doppler flow scans, was performed at 20 weeks’ gestation. Each participant and her newborn were seen by a research midwife in the early post-partum periods; neonatal length (centimeters (cm), *n* = 5289), weight (grams, *n* = 5609) and head circumference (cm, *n* = 5464) were measured within 72 h of birth. Neonatal length was measured using the neonatometer (*n* = 3171, 60.0%) or using tape measures to 0.1 of a centimeter [31]. Head circumference was measured with tape measures to 0.1 of a centimeter. Participants were asked about vaginal bleeding, infections, medication and supplement use during the 3rd trimester. Additional details of late pregnancy and delivery were collected from clinical case notes.

### Outcome

SGA was defined as birthweight less than the 10th customized birthweight percentile. Customized birthweight percentiles are adjusted for maternal booking weight, height, ethnicity, parity, gestational age, and sex of the infant using the Gestation Related Optimal Weight (GROW) software on www.gestation.net [2]. This software has been studied and found to be reliable in the detection of SGA with an increased risk of adverse perinatal outcome within multi ethnic populations and maternal under- and overweighted populations [32, 33]. SGA infants were grouped based on sex (males/females) and growth symmetry (symmetric/asymmetric). Asymmetric growth was defined as a Ponderal index < 10th percentile, corrected for gestational age based on reference values of Roje et al. [19]. Ponderal Index was calculated as (weight (grams) × 100)/(length (cm))^3^. Pregnancies were classified as uncomplicated in the absence of SGA, spontaneous and iatrogenic preterm birth, stillbirth, preeclampsia, gestational hypertension or gestational diabetes [34, 35].

### Statistical methods

Univariate analyses were performed for maternal demographics, pregnancy characteristics and neonatal outcome. For continuous variables, mean and median were compared using the Students *t* test and Mann-Whitney *U* test. Categorical variables were compared using chi-square test. Overall, less than 2% of the data was missing, 3 variables had > 5% missing data: maternal birthweight (5.2%), mean uterine Doppler resistance index (RI) (6.1%) and Ponderal Index (6.0%). For multivariable analysis, missing data were imputed using multiple imputation [36]. Multivariable analysis was performed using backward stepwise logistic regression to compare pregnancies complicated with SGA to uncomplicated pregnancies. SPSS default values (PIN = 0.05 and OUT = 0.1) were selected for the backward stepwise logistic regression.

Twenty-nine variables that were found to be associated with SGA in prior SCOPE publications by McCowan et al. and Khashan et al. were included in the multivariable analysis [7, 8, 37]. These variables are reported in Table S1. Following the stepwise procedure, 16 of the 29 variables were significantly associated with SGA and were included in the final model. The same 16 variables were included in the sex- and growth-specific multinominal multivariable analysis, with uncomplicated pregnancies as reference group. Percentages of missing data for each of these variables are shown in supplementary data (Table S2). The reported odds ratios (OR) of the multivariable analysis are pooled effects of the multiple imputation data, and were compared between the SGA subgroups. The threshold for significance was set at *p* < 0.05. Statistical analyses were performed using SPSS version 24 (SPSS Inc. Chicago, IL, USA).

## Results

**Fig. 1:**
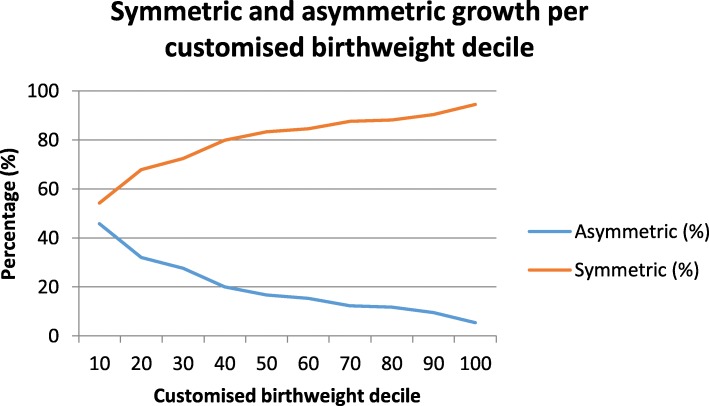
Percentage of symmetric and asymmetric growth per birthweight decile

A total of 5690 participants were enrolled in the SCOPE study of whom 62 (1.1%) participants were lost to follow-up or had a miscarriage or termination before 20 weeks’ gestation. Of the remaining 5628 pregnancies, 3376 (60.0%) were uncomplicated and 633 (11.2%) were complicated by SGA. Participant distribution per study site is shown in supplementary data (Table S3). Maternal demographics, pregnancy characteristics and outcome for the whole SCOPE cohort, uncomplicated and SGA pregnancies are presented in Table 1. Compared to uncomplicated pregnancies, women with a SGA pregnancy more frequently had a low birthweight themselves (*p* < 0.001), BMI < 20 or ≥ 30 (*p* < 0.001), a lower SEI score (*p* < 0.001) and higher systolic and diastolic blood pressure (*p* < 0.001) at 15± weeks’. Women with a SGA pregnancy were less likely to be Caucasian (*p* = 0.028) and less likely to have a Rhesus negative blood group (*p* = 0.022) compared with women with uncomplicated pregnancies. At 20 weeks’ gestation, women with a SGA pregnancy were more likely to smoke cigarettes and had higher uterine and umbilical Doppler flow RI compared to women with uncomplicated pregnancies (*p* < 0.001). The prevalence of asymmetric and symmetric growth by customized birthweight deciles for the SCOPE cohort are presented in Fig. 1. Of the SGA infants, 45.8% were asymmetric, compared to 5.5% of the infants with a birthweight > 90th percentile. The prevalence of symmetric and asymmetric growth was significantly different between customized birthweight deciles (*p* < 0.001). Within the whole SCOPE cohort, 606 males (22.5%) had asymmetric growth compared to 478 (18.4%) females (*p* < 0.001), these numbers include both SGA and non-SGA infants.

**Table 1:** Maternal demographics, pregnancy characteristics, and outcome

	All participants	Uncomplicated	SGA	*p* value	*%* missing
	*n* = 5628	*n* = 3376	*n* = 633		
Pre-pregnancy					
Maternal birthweight (g)	3308 (547)	3350 (529)	3170 (526)	0.000	5.2
Leafy vegetable intake ≥ 3/day	337 (6.0)	239 (7.1)	18 (2.8)	0.000	0
Fruit intake ≤ 1/week	500 (8.9)	250 (7.4)	83 (13.1)	0.000	0
15 weeks’ gestation					
Maternal age (years)	29 [25–32]	30 [25–33]	29 [24–33]	0.336	0
Maternal head circumference (cm)	55.7 (1.7)	55.8 (1.7)	55.5 (1.8)	0.000	0.2
Ethnicity				0.028	0
Caucasian	5061 (89.9)	3059 (90.6)	564 (89.1)		
Asian	170 (3.0)	110 (3.3)	16 (2.5)		
Indian	134 (2.4)	65 (1.9)	19 (3.0)		
African	65 (1.2)	27 (0.8)	8 (1.3)		
Other	198 (3.5)	115 (3.4)	26 (4.1)		
BMI				0.000	0.9
< 20	429 (7.6)	242 (7.2)	56 (8.9)		
20–25	2809 (49.9)	1842 (55.1)	272 (43.1)		
25.1–29.9	1500 (26.7)	860 (25.7)	183 (29.0)		
≥ 30	842 (15.0)	398 (11.9)	120 (19.0)		
Mean arterial pressure (mmHg)	79 (7.8)	78 (7.2)	80 (8.5)	0.000	0
Systolic blood pressure (mmHg)	107 [100–113]	105 [99–111]	108 [100–115]	0.000	0
Diastolic blood pressure (mmHg)	64 [60–70]	63 [60–69]	65 [60–72]	0.000	0
Random glucose (mmol/l)	5.3 (1.0)	5.3 (0.9)	5.2 (0.9)	0.064	1.3
Rhesus negative blood group	838 (14.9)	510 (15.1)	73 (11.6)	0.022	0.2
Socioeconomic index	45 [28–70]	45 [29–50]	43 [27–50]	0.000	0
Daily vigorous exercise	54 (1.0)	25 (0.7)	14 (2.2)	0.001	0.4
20 weeks’ data					
Smoking > 15 weeks' gestation	607 (10.8)	309 (9.2)	121 (19.1)	0.000	0
Uterine Doppler mean RI	0.57 (0.10)	0.56 (0.10)	0.61 (0.11)	0.000	6.1
Umbilical Doppler RI	0.73 (0.06)	0.73 (0.06)	0.74 (0.07)	0.000	4.1
Umbilical Doppler RI > 90^th^ percentile	516 (9.2)	238 (8.6)	109 (17.2)	0.000	4.1
Ultrasound HC/AC > 95th percentile	273 (4.9)	153 (4.6)	45 (7.2)	0.006	2.3
Perceived stress score	12 (6.5)	12 (6.4)	13 (6.3)	0.001	3.1
Pregnancy outcome					
Pre-eclampsia	374 (6.6)	0 (0.0)	166 (26.2)	0.000	0
Gestational diabetes	143 (2.5)	0 (0.0)	10 (1.6)	0.000	0.3
Neonatal characteristics					
Birthweight (g)	3401 (591.5)	3594 (398.6)	2608 (578.0)	0.000	0.3
Customized birthweight percentile	47.6 (29.1)	54.2 (25.2)	4.7 (3.0)	0.000	0.4
Gestational age (days)	277 (17.7)	281 (8.1)	272 (24.5)	0.000	0
Spontaneous preterm birth (< 37 weeks)	236 (4.2)	0 (0.0)	26 (4.1)	0.000	0
Ponderal index (g/m^3^)	2.68 [2.48–2.88]	2.71 [2.53–2.89]	2.45 [2.28–2.62]	0.000	6.0
Ponderal index < 10th percentile for gestation	1084 (19.3)	565 (17.6)	262 (45.8)	0.000	6.0
Stillbirth	37 (0.7)	0 (0.0)	20 (3.2)	0.000	0.3

All values are mean (SD) and median [IQR] for continuous variables and absolute numbers (percentages) for categorical variables. *BMI* body mass index (calculated as weight in kilograms divided by height in meters squared). *RI* resistance index (calculated as peak systolic flow minus end diastolic flow divided by peak systolic flow). *HC/AC* head circumference to abdominal circumference ratio

Maternal demographics, pregnancy characteristics, and outcome for SGA by fetal sex and growth symmetry are presented in Table 2. Between male and female SGA infants, there were no significant differences in maternal demographics or clinical risk factors at 15 weeks’ gestation. However, maternal SEI was on average lower for women bearing a female SGA infant compared to those bearing a male SGA infant (37 [26–50] vs 45 [28–50], *p* = 0.054). At 20 weeks’ gestation, mean umbilical Doppler RI was significantly different between male and female SGA-bearing pregnancies (0.73 vs 0.75, *p* = 0.003). Regarding neonatal outcome, SGA males had a lower Ponderal Index compared to female SGA infants (2.42 vs 2.48, *p* = 0.013) and thus had more often an asymmetric growth pattern (51.2% vs 40.4%, *p* = 0.009).

**Table 2:** Univariate analysis at 15 and 20 weeks' gestation and after delivery in SGA infants

	Males *N* = 313	Females *N* = 320	*p* value	Asymmetric *N* = 262	Symmetric *N* = 310	*p* value	% missing
Pre-pregnancy							
Maternal birthweight (g)	3160 (568)	3179 (483)	0.209	3148 (542)	3167 (527)	0.680	6.5
Leafy vegetable intake ≥ 3/day	11 (3.5)	7 (2.2)	0.315	11 (4.2)	6 (1.9)	0.112	0
Fruit intake ≤ 1/week	38 (12.1)	45 (14.1)	0.474	29 (11.1)	46 (14.8)	0.183	0
15 weeks’ gestation							
Maternal age	29 (5.8)	28 (2.8)	0.075	29 (5.4)	28 (6.0)	0.286	0
Maternal head circumference (cm)	55.4 (1.8)	55.6 (1.8)	0.363	55.4 (1.8)	55.6 (1.8)	0.370	0.2
Ethnicity			0.425			0.001	0
Caucasian	277 (88.5)	287 (89.7)		226 (86.3)	284 (91.6)		
Asian	9 (2.9)	7 (2.2)		11 (4.2)	5 (1.6)		
Indian	10 (3.2)	9 (2.8)		13 (5.0)	2 (0.6)		
African	3 (1.0)	5 (1.6)		5 (1.9)	1 (0.3)		
Other	14 (4.5)	12 (3.8)		7 (2.7)	18 (5.8)		
BMI			0.735			0.030	0.3
< 20	31 (9.9)	25 (7.8)		20 (7.6)	28 (9.1)		
20–25	130 (41.7)	142 (44.5)		124 (47.3)	124 (40.3)		
25.1–29.9	93 (29.8)	90 (28.2)		81 (30.9)	84 (23.4)		
≥ 30	58 (18.6)	62 (19.2)		37 (14.1)	72 (23.4)		
Mean arterial pressure (mmHg)	81 (8.8)	80 (8.2)	0.548	80 (8.4)	81 (8.6)	0.427	0
Systolic blood pressure (mmHg)	109 [11.0]	108 [10.6]	0.612	108 [10.0]	109 [11.6]	0.348	0
Diastolic blood pressure (mmHg)	67 [8.8]	66 [8.1]	0.561	66 [8.9]	67 [8.3]	0.553	0
Random glucose	5.4 (0.9)	5.2 (1.0)	0.194	5.2 (1.0)	5.2 (0.9)	0.796	1.6
Rhesus negative blood group	38 (12.3)	35 (11.0)	0.604	28 (10.8)	37 (12.0)	0.654	0.8
Socioeconomic index	45 [28–50]	37 [26–50]	0.054	45 [29–50]	36 [22–50]	0.010	0
Daily vigorous exercise	8 (2.6)	6 (1.9)	0.564	3 (1.1)	9 (2.9)	0.138	0.8
20 weeks’ gestation							
Smoking > 15 weeks gestation	56 (17.9)	65 (20.3)	0.439	46 (17.6)	68 (21.9)	0.192	0
Uterine Doppler RI	0.61 (0.11)	0.60 (0.11)	0.081	0.60 (0.11)	0.61 (0.11)	0.338	7.1
Umbilical Doppler RI	0.73 (0.06)	0.75 (0.07)	0.003	0.74 (0.07)	0.74 (0.06)	0.299	4.3
Umbilical Doppler RI > 90th percentile	29 (9.6)	54 (17.8)	0.003	34 (13.4)	41 (13.9)	0.888	4.3
Ultrasound HC/AC > 95th percentile	22 (7.2)	23 (7.3)	0.966	16 (6.2)	24 (7.9)	0.444	1.7
Perceived stress score	13 (6.4)	13 (6.3)	0.888	14 (6.5)	13 (6.3)	0.051	3.5
Pregnancy outcome							
Pre-eclampsia	85 (27.2)	81 (25.3)	0.598	74 (28.2)	81 (26.1)	0.571	0
Gestational diabetes	5 (1.6)	5 (1.6)	0.513	5 (1.9)	5 (1.6)	0.962	0
Induction of labour	120 (38.3)	110 (34.4)	0.300	91 (34.7)	122 (39.4)	0.255	0
Emergency caesarean section	56 (8.8)	44 (7.0)	0.153	43 (7.5)	54 (9.4)	0.749	0
Neonatal characteristics							
Birthweight (g)	2780 [2483—2990]	2720 [2433—2970]	0.112	2745 [2474—2970]	2780 [2438—2986]	0.961	0
Customized birthweight percentile	4.8 (3.0)	4.7 (3.0)	0.694	4.4 (2.8)	5.0 (3.0)	0.018	0
Gestational age (days)	272 (24.1)	272 (25.0)	0.872	276 (15.6)	270 (26.4)	0.003	0
Spontaneous preterm birth (< 37 weeks)	10 (3.2)	16 (5.0)	0.253	6 (2.3)	17 (5.5)	0.053	0
Ponderal Index	2.42 [2.24—2.59]	2.48 [2.30—2.64]	0.013	2.21 [2.10 - 2.37]	2.60 [2.51—2.72]	0.000	9.6
Ponderal Index < 10th percentile for gestation	147 (51.2)	115 (40.4)	0.009				9.6
Head circumference (cm)	33.2 (2.7)	32.8 (2.4)	0.000	33.3 (2.1)	32.7 (2.8)	0.010	5.4
Length (cm)	48.5 [46.5–50.0]	48.0 [46.0–49.0]	0.004	49.35 [48.0–51.0]	46.8 [45.1–48.3]	0.000	9.6
Male				147 (56.1)	140 (45.2)	0.009	0
Stillbirth	9 (2.9)	11 (3.4)	0.686	2 (0.8)	10 (3.2)	0.041	0
5-min Apgar < 7	7 (2.3)	8 (2.6)	0.829	3 (1.2)	11 (3.7)	0.060	4.4
Neonatal death	0 (0.0)	1 (0.3)	0.322	0 (0.0)	0 (0.0)	n/a	0
Admitted to nursery	80 (25.6)	63 (19.7)	0.077	56 (24.1)	75 (24.2)	0.424	0

All values are mean (SD) and median [IQR] for continuous variables and absolute numbers (percentages) for categorical variables. *BMI* body mass index (calculated as weight in kilograms divided by height in meters squared). *RI* resistance index (calculated as peak systolic flow minus end diastolic flow divided by peak systolic flow). *HC/AC* head circumference to abdominal circumference ratio

Compared to asymmetric SGA, women bearing a symmetric SGA infant were more often Caucasian (*p* = 0.001), more often had a BMI < 20 or ≥ 30 (*p* = 0.030) and had lower SEI scores (36 [22–50] for symmetric and 45 [29–50] for asymmetric SGA, *p* = 0.010). There were no significant differences between symmetric and asymmetric SGA infants in mean umbilical and uterine Doppler RI at 20 weeks’ gestation. Regarding neonatal outcome, asymmetric SGA infants had a lower customized birthweight percentile compared to symmetric SGA (mean of 4.4 (2.8) and 5.0(3.0) respectively, *p* = 0.017). Symmetric SGA infants were more often born spontaneously pre-term (< 37 weeks) than asymmetric SGA infants (5.5% vs 2.3%, *p* = 0.053).

Table 3 shows the OR of clinical risk factors with a significant independent association with SGA, compared to uncomplicated pregnancies. Separate analyses were performed for the SGA subgroups of interest. Daily vigorous exercise was significantly associated with both SGA males (4.2 (1.8–10.0)) and SGA females (2.7 (1.1–7.1)). The OR per unit increase for Uterine Doppler RI was higher in SGA males (1.7 (1.5–1.9)) than females (1.5 (1.3–1.7)). Whereas for Umbilical Doppler RI this was only significantly associated with SGA females (1.6 (1.3–1.9) vs 1.0 (0.8–1.3)). In sensitivity analyses, we restricted multivariate testing to unimputed data excluding missing data (Table S4). These showed similar results to multivariate testing with imputed data.

**Table 3:** Multivariate comparisons of SGA and SGA subgroups compared to uncomplicated pregnancies

	All SGA	Male SGA	Female SGA	Asymmetric SGA	Symmetric SGA
	*n* = 633	*n* = 313	*n* = 320	*n* = 262	*n* = 310
Pre-pregnancy					
Maternal birthweight ↓ 200 gr	**1.1 (1.1–1.2)**	**1.1 (1.1–1.2)**	**1.1 (1.1–1.2)**	**1.1 (1.1–1.2)**	**1.1 (1.1–1.2)**
Leafy veg intake pre-pregnancy 3/day	**0.5 (0.3–0.8)**	0.6 (0.3**–**1.1)	**0.4 (0.2–0.8)**	0.7 (0.4**–**1.3)	**0.3 (0.1–0.7)**
Fruit intake pre-pregnancy ≤ 1/week	**1.5 (1.1–2.0)**	**1.5 (1.0–2.2)**	**1.6 (1.1–2.2)**	1.4 (0.9**–**2.1)	**1.7 (1.2–2.5)**
15 weeks’ gestation					
Maternal age ↑ 5 years	**1.1 (1.0–1.2)**	**1.2 (1.1–1.4)**	1.0 (1.0**–**1.2)	**1.2 (1.1–1.4)**	1.2 (1.0**–**1.3)
Maternal head circumference ↑ 1 cm	**0.9 (0.9–1.0)**	**0.9 (0.8–1.0)**	**0.9 (0.9–1.0)**	**0.9 (0.8–1.0)**	**0.9 (0.9–1.0)**
Maternal BMI ↑ 5 units	**1.2 (1.1–1.3)**	1.1 (1.0**–**1.3)	**1.2 (1.1–1.4)**	1.1 (0.9**–**1.2)	**1.3 (1.1–1.5)**
Mean arterial pressure ↑ 5 units mmHg	**1.2 (1.2–1.3)**	**1.3 (1.2–1.4)**	**1.2 (1.1–1.3)**	**1.2 (1.1–1.4)**	**1.2 (1.1–1.3)**
Binge drinking or recreational drug use	**1.4 (1.1–1.7)**	1.3 (1.0**–**1.7)	**1.5 (1.1–1.9)**	1.3 (1.0**–**1.8)	1.2 (0.9**–**1.5)
Rhesus negative blood group	**0.8 (0.6–1.0)**	0.8 (0.6**–**1.2)	0.7 (0.5**–**1.0)	0.7 (0.5**––**1.1)	0.8 (0.6**–**1.1)
Random glucose ↑ 1 unit	**0.9 (0.8–1.0)**	**0.8 (0.7–1.0)**	0.9 (0.8**–**1.1)	0.9 (0.8**–**1.1)	**0.9 (0.8–1.0)**
Daily vigorous exercise	**3.4 (1.6–7.1)**	**4.2 (1.8–10.0)**	**2.7 (1.1–7.1)**	1.6 (0.5–5.7)	**4.4 (1.9–10.3)**
Tertiary student	**2.0 (1.2–3.2)**	**2.4 (1.3–4.3)**	1.6 (0.8**–**3.1)	**2.5 (1.3–4.6)**	1.8 (0.9**–**3.6)
20 weeks’ gestation					
Smoking > 15 weeks’ gestation	**1.8 (1.4–2.4)**	**1.9 (1.4–2.8)**	**1.8 (1.2–2.5)**	**1.9 (1.3–2.8)**	**2.2 (1.5–3.1)**
Perceived stress score at 20 weeks’ ↑ 5	**1.1 (1.0–1.2)**	1.1 (1.0**–**1.2)	1.1 (1.0**–**1.2)	**1.2 (1.1–1.3)**	1.0 (0.4**–**1.1)
Uterine Doppler mean RI ↑0.1	**1.6 (1.4–1.7)**	**1.7 (1.5–1.9)**	**1.5 (1.3–1.7)**	**1.5 (1.3–1.7)**	**1.6 (1.4–1.8)**
Umbilical Doppler RI ↑0.1	**1.3 (1.1–1.5)**	1.0 (0.8**–**1.3)	**1.6 (1.3–1.9)**	1.2 (1.0**–**1.5)	**1.4 (1.1–1.7)**

Results are expressed as OR (95%CI) with uncomplicated pregnancies as the referent group. Bold indicate that the OR is significant

Daily vigorous exercise (4.4 (1.9–10.3), low fruit intake (1.7 (1.2–2.5)), and high leafy vegetable intake (0.3 (0.1–0.7)) were significantly associated with symmetric SGA, but not with asymmetric SGA. Perceived stress score at 20 weeks’ gestation only had a significant association with asymmetric SGA (1.2 (1.1–1.3)).

## Discussion

### Main findings

The data from this large prospective cohort demonstrate that there is a substantial variance in risk factors and neonatal outcome for SGA based on fetal sex and growth symmetry. Low birthweight percentiles and male sex are associated with higher rates of asymmetric growth.

In the present study, we did not find significant sex-specific differences in pregnancy outcome, regarding stillbirth, low Apgar scores, and preeclampsia. SGA males were generally longer and had a relatively larger head circumference but were not heavier than SGA females. Asymmetric growth was predominantly seen in SGA males, while symmetric growth was more commonly seen in females, implying that growth trajectory, specifically growth symmetry, is sex-specific.

Previous research showed that the predictive value of HC/AC ratio is low and poorly correlated with Ponderal Index and should therefore be rejected as a measurement for asymmetric growth *in utero* [14, 18, 38]. This is consistent with the present study, where the rates of infants with a HC/AC ratio > 95th percentile at the time of the 20 weeks’ morphology scan were not significantly different between symmetric and asymmetric SGA infants. One might speculate that the fetus demonstrating HC/AC discordance is more easily recognised by ultrasound compared to the symmetrically growing fetus. However, the rate of induction of labour or emergency caesarean section was not different between the two SGA groups. Compared to symmetric SGA, asymmetric SGA infants had lower customized birthweight percentiles, but were longer and had a relatively larger head circumference, suggesting potential brain sparing.

### Interpretation

Our findings are consistent with the theories reported by Resnik et al. and Clifton et al. that symmetric growth restriction occurs earlier in pregnancy than asymmetric growth restriction and that there are sex-specific strategies by which males and females cope with adverse in utero environments [15, 23].

Symmetric growth restriction is hypothesized to be caused by early whole body impairment of fetal growth, for example, by maternal drug use, infection or chromosomal abnormalities [15]. In contrast, asymmetric growth restriction may arise later in gestation, due to inadequate availability of substrates for fetal growth possibly caused by maternal vascular disease and decreased uteroplacental perfusion [15]. In the SCOPE cohort, clinical risk factors for SGA, such as low and high maternal BMI, low SEI, and pre-pregnancy diet, seem to be more strongly associated with symmetric SGA. However, importantly in the present study increased uterine artery Doppler RI and preeclampsia were not more prevalent within asymmetric SGA pregnancies. In contrast with previous findings, asymmetric SGA infants had lower rates of stillbirth, spontaneous preterm birth, and higher Apgar scores compared to symmetric SGA infants [20].

Most studies report no negative consequences of (vigorous) exercise during pregnancy on fetal well-being [39, 40]. Clapp et al. reported improved fetoplacental growth in women who begin or maintain exercise in early pregnancy and decrease their exercise in mid and late pregnancy [41, 42]. However, McCowan et al. found daily vigorous exercise as a major risk factor for SGA [7]. The present study can add to this that the association between vigorous exercise in early pregnancy and SGA may be stronger for male SGA than female SGA infants.

Zhou et al. reported a gene environment interaction for the maternal angiotensin-converting enzyme (ACE) A11860G gene variant and low SEI or low leafy vegetable intake as a risk factor for SGA in female-bearing pregnancies. ACE gene encodes a potent zinc metalloenzyme involved in renin-angiotensin system (RAS) activity which is also involved in the trophoblast function [43]. Myatt et al. studied trophoblast function in placentas of obese women and reported reduced mitochondrial respiration and adenosine triphosphate (ATP) generation [44]. Findings of both studies suggest compromised placental function. In the present study, female and symmetric SGA were both associated with low leafy vegetable intake and increased maternal BMI. The increased umbilical Doppler RI in these SGA subgroups suggests impaired growth of the placenta over the first 20 weeks’. Maternal BMI, leafy vegetable intake and umbilical Doppler RI were not significantly associated with male and asymmetric SGA, suggesting that these SGA subgroups may have a different pathogenesis.

### Strengths and limitations

To our knowledge, the present study is the first to report on growth symmetry and sex differences in SGA infants. The strength of this prospective study is the extensive amount of detailed information before and during pregnancy. The limitation is that, while this is a large prospective cohort study, the number of SGA infants (*n* = 633) is insufficient to investigate stillbirth and neonatal death rates and thus these findings should be interpreted with caution in a clinical context. Furthermore, the neonatometer was used for 60.0% of the neonates, the remaining 40.0% of the neonates were measured with a tape measure. This may have introduced variability in length measurements. Maternal weight gain was not included in the study design of the SCOPE study; therefore, we are unable to provide any details regarding maternal weight gain. Although the GROW software has been found to be reliable in the detection of SGA with an increased risk of adverse perinatal outcome within multi ethnic populations and maternal under- and overweight populations, the use of customized birthweight centiles to determine SGA infants is not universally accepted [32, 33].

## Conclusion

Among SGA infants low customized birthweight percentiles and male sex are associated with asymmetric SGA. Poor maternal health in early pregnancy is associated with symmetric SGA, while increased uterine Doppler flow in later pregnancy is associated with both symmetric and asymmetric SGA. Further research regarding the biology of growth symmetry and the value of additional Doppler flow scans as predictors of growth symmetry may aid in a better insight in the pathophysiology of different SGA phenotypes.

### Perspectives and significance

This manuscript contributes to an improved understanding of the aetiology of sex-specific strategies by which males and females cope with adverse in utero environments. We demonstrate that there is a substantial variance in risk factors and neonatal outcome for SGA based on fetal sex and growth symmetry. Among SGA infants, low birthweight percentiles and male sex are associated with higher rates of asymmetric growth which has different risk factors compared to symmetric fetal growth, indicating a different pathogenesis.

## Supplementary information

**Additional file 1: Table S1–S4.**

## Data Availability

The data that support the findings of this study are available from the SCOPE Consortium but restrictions apply to the availability of these data, which were used under license for the current study, and so are not publicly available. Data are however available from the authors upon reasonable request and with permission of the SCOPE Consortium.
