# Supplementary material for: Sex- and growth-specific characteristics of small for gestational age infants: a prospective cohort study
Source: Biol Sex Differ. 2020 May 5;11:25. doi: 10.1186/s13293-020-00300-z (PMC7201715; doi:10.1186/s13293-020-00300-z)
Supplement: Supplementary file 1 — Additional file 1: Table S1–S4. [file 13293_2020_300_MOESM1_ESM.docx]

Supplement 1

|  | **Variable** |
| --- | --- |
| 1 | Strong family history of preeclampsia |
| 2 | Recreational drug use at 15 wks |
| 3 | Conception by *in vitro* fertilisation |
| 4 | Green leafy vegetable intake <3 servings per day in the month prior to pregnancy. |
| 5 | Low fruit intake (<1 piece of fruit per wk) in the month prior to pregnancy. |
| 6 | Socioeconomic index |
| 7 | Average umbilical RI (per 0.1 unit) |
| 8 | Mean arterial pressure (per 5 unit) |
| 9 | Maternal head circumference (per 1 cm) |
| 10 | Previous pregnancy before 20 weeks |
| 11 | Ethnicity |
| 12 | Tertiary education student |
| 13 | Maternal age (per 5 years) |
| 14 | Maternal birthweight (per 200g) |
| 15 | Body mass index |
| 16 | Rhesus negative blood group |
| 17 | Daily vigorous exercise |
| 18 | Single |
| 19 | No paid work |
| 20 | Smoking at 15 weeks |
| 21 | Average Uterine Doppler RI (per 0.1 unit) |
| 22 | Recreational walking ≥ 4 x/week |
| 23 | Random glucose (per 1 unit_ |
| 24 | >12 months to conceive |
| 25 | Anxiety score at 15 weeks pregnant |
| 26 | Depression score at 15 weeks pregnant |
| 27 | Anxiety score at 20 weeks pregnant |
| 28 | Perceived stress score at 20 weeks pregnant |
| 29 | Depression score at 20 weeks pregnant |

1-21 McCowan, L. M. E., et al. "Risk factors for small‐for‐gestational‐age infants by customised birthweight centiles: data from an international prospective cohort study." *BJOG: An International Journal of Obstetrics & Gynaecology* 117.13 (2010): 1599-1607.

13-24 McCowan, Lesley ME, et al. "Clinical prediction in early pregnancy of infants small for gestational age by customised birthweight centiles: findings from a healthy nulliparous cohort." *PLoS One* 8.8 (2013): e70917.

25-29 Khashan, A. S., et al. "Second-trimester maternal distress increases the risk of small for gestational age." *Psychological medicine* 44.13 (2014): 2799-2810.

Supplement 2

|  | Uncomplicated | | SGA | |
| --- | --- | --- | --- | --- |
|  | N available | N missing (%) | N available | N missing (%) |
| **Pre pregnancy** |  |  |  |  |
| Maternal birthweight ↓ 200 gr | 3223 | 153 (4.5) | 592 | 41 (6.5) |
| Leafy vegetable intake pre-pregnancy 3/day | 3376 | 0 (0.0) | 633 | 0 (0.0) |
| Fruit intake pre-pregnancy ≤1/week | 3376 | 0 (0.0) | 633 | 0 (0.0) |
| **First visit at 15 weeks gestation** |  |  |  |  |
| Maternal age ↑ 5 years | 3376 | 0 (0.0) | 633 | 0 (0.0) |
| Maternal head circumference ↑ 1 cm | 3371 | 5 (0.1) | 632 | 1 (0.2) |
| Maternal BMI ↑ 5 | 3376 | 0 (0.0) | 633 | 0 (0.0) |
| Mean arterial pressure ↑ 5 | 3376 | 0 (0.0) | 633 | 0 (0.0) |
| Binge drinking or recreational drug use 1st trimester | 3376 | 0 (0.0) | 633 | 0 (0.0) |
| Rhesus negative blood group | 3371 | 5 (0.1) | 628 | 5 (0.8) |
| Random glucose ↑ 1 unit increase | 3331 | 45 (1.3) | 623 | 10 (1.6) |
| Daily vigorous exercise | 3361 | 15 (0.4) | 628 | 5 (0.8) |
| Tertiary student | 3376 | 0 (0.0) | 633 | 0 (0.0) |
| **Second visit at 20 weeks gestation** |  |  |  |  |
| Smoking > 15wks gestation | 3376 | 0 (0.0) | 633 | 0 (0.0) |
| Perceived stress score at 20 weeks ↑ 5 | 3285 | 91 (2.7) | 611 | 22 (3.5) |
| Uterine Doppler mean RI ↑0.1 | 3194 | 182 (5.4) | 588 | 45 (7.1) |
| Umbilical Doppler RI ↑0.1 | 3258 | 118 (3.5) | 606 | 27 (4.3) |

**Supplement 3**

**Participant distribution per site**

|  | **United Kingdom** | **New Zealand** | **Ireland** | **Australia** |
| --- | --- | --- | --- | --- |
| **Participants** | **658** | **2032** | **1774** | **1164** |
| **Uncomplicated pregnancy (%)** | **379 (57.6)** | **1376 (67.7)** | **1013 (57.1)** | **608 (52.2)** |
| **Pregnancy complicated by SGA (%)** | **101 (15.3)** | **201 (9.9)** | **190 (10.7)** | **141 (12.1)** |
| **Pregnancy complicated by other than SGA (%)** | **178 (27.1)** | **455 (22.4)** | **571 (32.2)** | **415 (35.7)** |

Supplement 4

|  | **All SGA** | **Male SGA** | **Female SGA** | **Asymmetric SGA** | **Symmetric SGA** |
| --- | --- | --- | --- | --- | --- |
|  | **n = 519** | **n = 260** | **n = 266** | **n =262** | **n = 217** |
| **Pre-pregnancy** |  |  |  |  |  |
| Maternal birthweight ↓ 200 gr | **1.2 (1.1 – 1.2)** | **1.1 (1.1 - 1.2)** | **1.1 (1.1 - 1.2)** | 1.2 (0.8 - 1.2) | **1.2 (1.1 - 1.2)** |
| Leafy veg intake pre-pregnancy 3/day | **0.5 (0.3 - 0.9)** | 0.4 (0.3 - 1.3) | **0.4 (0.2 - 0.9)** | 0.8 (0.4 – 1.4) | **0.4 (0.2 – 0.9)** |
| Fruit intake pre-pregnancy ≤1/week | **1.5 (1.1 - 2.0)** | 1.3 (0.8 - 2.0) | **1.6 (1.1 - 2.4)** | 1.1 (0.7 – 1.8) | **1.8 (1.2 - 2.7)** |
| **15 weeks’ gestation** |  |  |  |  |  |
| Maternal age ↑ 5 years | **1.1 (1.1 – 1.2)** | **1.2 (1.1 - 1.4)** | 1.1 (0.9 - 1.2) | 1.2 (1.0 - 1.4) | **1.2 (1.1 - 1.4)** |
| Maternal head circumference ↑ 1 cm | **0.9 (0.9 - 1.0)** | **0.9 (0.8 – 0.9)** | 0.9 (0.9 - 1.0) | **0.9 (0.8 – 0.9)** | **0.9 (0.8 - 1.0)** |
| Maternal BMI ↑ 5 units | **1.2 (1.0 – 1.3)** | **1.2 (1.0 - 1.4)** | **1.2 (1.0 - 1.4)** | 1.1 (0.9 - 1.3) | **1.3 (1.1 - 1.5)** |
| Mean arterial pressure ↑ 5 units mmHg | **1.2 (1.1 – 1.3)** | **1.3 (1.1 - 1.4)** | **1.2 (1.1 - 1.3)** | **1.2 (1.1 - 1.4)** | **1.3 (1.2 - 1.4)** |
| Binge drinking or recreational drug use | **1.4 (1.1 - 1.7)** | 1.2 (0.9 - 1.7) | **1.5 (1.1 - 1.9)** | 1.3 (0.9 - 1.8) | 1.1 (0.9 - 1.8) |
| Rhesus negative blood group | **0.8 (0.6 - 1.0)** | 0.8 (0.5 - 1.2) | 0.7 (0.5 - 1.1) | 0.7 (0.4 - 1.1) | 0.8 (0.5 - 1.2) |
| Random glucose ↑ 1 unit | **0.9 (0.8 - 1.0)** | **0.8 (0.7 - 1.0)** | 0.9 (0.8 - 1.1) | 0.9 (0.8 – 1.1) | 0.9 (0.8 - 1.0) |
| Daily vigorous exercise | **2.4 (1.0 – 5.6)** | **3.0 (1.0 – 8.8)** | 2.6 (0.9 - 7.2) | 0.6 (0.1 – 4.8) | **3.7 (1.4 – 9.7)** |
| Tertiary student | **1.9 (1.1 - 3.4)** | **2.6 (1.3 – 5.2)** | 1.4 (0.6 - 3.1) | **2.5 (1.2 – 5.1)** | 1.8 (1.0 – 1.3) |
| **20 weeks’ gestation** |  |  |  |  |  |
| Smoking > 15 weeks’ gestation | **1.8 (1.4 - 2.5)** | **2.0 (1.4 – 3.0)** | **1.8 (1.2 - 2.5)** | **1.9 (1.2 – 2.9)** | **2.3 (1.6 – 3.4)** |
| Perceived stress score at 20 weeks’ ↑ 5 | **1.0 (1.0 – 1.3)** | 1.1 (1.0 - 1.2) | 1.1 (1.0 - 1.2) | **1.2 (1.1 - 1.3)** | 1.0 (0.9 - 1.1) |
| Uterine Doppler mean RI ↑0.1 | **1.5 (1.4 – 1.7)** | **1.4 (1.2 - 1.6)** | **1.5 (1.3 - 1.7)** | **1.3 (1.2 - 1.5)** | **1.6 (1.4 - 1.8)** |
| Umbilical Doppler RI ↑0.1 | **1.3 (1.1 – 1.5)** | 1.2 (1.2 - 1.9) | **1.6 (1.3 - 1.9)** | 1.2 (1.0 - 1.5) | **1.3 (1.1 - 1.6)** |
